# Prospective Functions of miRNA Variants May Predict Breast Cancer Among Saudi Females

**DOI:** 10.7759/cureus.47849

**Published:** 2023-10-28

**Authors:** Samar N Ekram, Ghydaa Alghamdi, Abdelrahman N Elhawary, Hatem A Sembawa, Abdulsalam A Noorwali, Ikhlas A Sindi, Nasser A Elhawary

**Affiliations:** 1 Department of Medical Genetics, College of Medicine, Umm Al-Qura University, Mecca, SAU; 2 Department of Diabetes and Endocrinology, Queen Elizabeth Hospital Birmingham, Birmingham, GBR; 3 Department of Surgery, College of Medicine, Umm Al-Qura University, Mecca, SAU; 4 Department of Medicine, College of Medicine, Umm Al-Qura University, Mecca, SAU; 5 Department of Biotechnology, Faculty of Science, King Abdulaziz University, Jeddah, SAU

**Keywords:** case-control study, saudi females, protein-protein interactions, linkage analysis, taqman genotyping assay, microrna expression, breast cancer research

## Abstract

Background

Growing knowledge supports the importance of microRNAs (miRNAs) in modulating the initiation and development of breast cancer (BC) and underlying mechanisms. BC is a significant public health in females worldwide, where it remains the leading cause of death among Saudi females. Here, we evaluate the susceptibility of the miRNA genetic variants to the risk of BC in Saudi females.

Methods

One hundred fifty-four females, including 76 females diagnosed with BC and 78 healthy controls, were analyzed using TaqMan™ (Thermo Fischer Scientific, Waltham, MA) genotyping assays for the miR-196a2 rs11614913 C>T, miR-146a rs2910164 C>G, and miR-499 rs3746444 A>G. We utilized the SNPStats software (https://www.snpstats.net) (Institut Català d'Oncologia, Barcelona, Spain) to choose the best interactive inheritance model for the examined miRNAs.

Results

The examined miRNA single-nucleotide polymorphisms (SNPs) showed no clear association with the risk of BC (P > 0.05). As for genotypic distributions, significant associations were found for the rs2910164 SNP in most interactive models of inheritance: 2.50 (95% confidence interval {CI}, 1.2-5.17; P = 0.0135) in the codominant model, 2.34 (95% CI, 1.11-4.8; P = 0.0197) in the dominant model, and 2.40 (95% CI, 1.22-4.73; P = 0.0113) in overdominant model. The rs2910164 C/G heterozygosity showed overexpression in cases compared to controls (73.7% versus 53.9%; chi-squared (χ^2^) = 6.5; P = 0.0109), but the homozygous rs2910164 G/G showed a significant protective effect (21.1% versus 38.5%; χ^2^ = 17.4; P = 0.019). The heterozygosity did not affect the risk to the BC in the two miRNAs (rs11614913 C>T and rs3746444 A>G).

Conclusion

Despite lacking associations with the examined miRNAs, the heterozygous genotype rs2910164 C/G can identify at-risk females. More studies should be replicated using a panel of miRNA genes to discover significant associations with the risk of BC.

## Introduction

Breast cancer (BC) is the most dominant type of cancer, with about 2.261 million new female cases (11.7% of all cancers worldwide) in 2020. Despite advances in BC prognoses and treatments, metastasis remains the leading cause of mortality, with 684,996 per 100,000 deaths at age 85+, accounting for about 15.5% of 513,525 deaths before the age of 75 (GLOBOCAN 2020, https://gco.iarc.fr/today/). Epidemiological studies suggest that the incidence of BC continues to increase in most countries globally [1]. However, the worldwide incidence of female BC remains high, although the new cases have dramatically declined in most industrialized countries, except Western European countries and Australia/New Zealand [1].

According to GLOBOCAN (https://gco.iarc.fr/today/), powered by the World Health Organization, the new cases of female BC among Saudis are estimated at 27.5 per 100,000 (3,777 new cases), and the mortality rate (age-standardized mortality rate {ASMR}) is 7.7 per 100,000 females (977 deaths) diagnosed with BC. According to the classification criteria based on the origin of transformed cells (histological type), the most frequent histology is ductal, lobular, and mixed (containing lobular and ductal transformed cells). Blood and lymph vessels can spread cancer outside the breast, which has metastasized when it reaches other body parts [2].

Several risk factors associated with BC in females include early menarche age and delayed menopause, late age at first birth, hormonal intake, and obesity [3]. Otherwise, breastfeeding reduces a female's risk of BC and has considerable protective consequences [4]. Pathogenic penetrating variants in *BRCA1*, *TP53*, and *PALB2* genes, especially in young females aged <40 years [5], have been investigated for a long time and are risk factors for hereditary BC [6].

MicroRNAs (miRNAs) are a group of highly conserved small noncoding RNA molecules with 18-24 nucleotides [7]. They can target mRNA during gene expression by binding them to the 3' untranslated regions of mRNAs, leading to the degradation or repression of mRNAs [8]. Several publications revealed that miRNAs are extensively involved in cell proliferation, differentiation, and apoptosis [9,10]. However, miRNAs can function as oncogenes or tumor suppressors in different carcinomas, including BC [9,11,12]. miRNAs might act as biomarkers for the detection and prognosis of BC and treatment targets, providing endless possibilities for determining BC biomarkers and therapeutic strategies [13].

Few reports on miRNAs and the regulation of candidate genes involving the risk of BC are still inadequate in the Saudi population [14,15]. In addition, research on candidate gene miRNA polymorphisms, miR-196a2 (MIM 609687), miR-146a (MIM 610566), and miR-499 (MIM 613614), and their implications on BC risk is still insufficient. The present study has extendedly examined the potential effect of miR-146a rs2910164 G>C, miR-196a2 rs11614913 C>T, and miR-499 rs3746444 A>G single-nucleotide polymorphisms (SNPs) on the risk of BC in Saudi females.

## Materials and methods

Ethics statement and consent

All study participants signed informed consent. The study protocols were approved by the Institutional Biomedical Ethics Committee of Umm Al-Qura University (reference number: HAPO-02-K-012) and licensed by the National Committee of Medical and Bioethics, King Abdulaziz City for Science and Technology (KACST), Riyadh.

Study population

**Figure 1:**
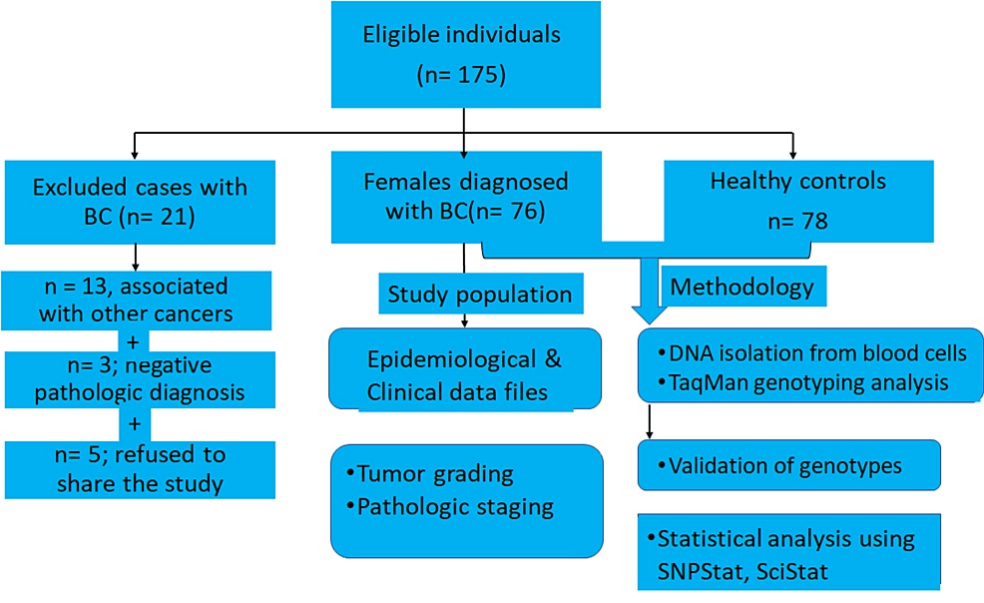
Flow chart of study inclusion and exclusion and applied methodology in females with breast cancer (BC)

The study was conducted on 76 Saudi females (average: 27-74 years) with BC who were referred to the Department of Genetic Oncology at King Abdullah Medical City Hospital, Mecca, between October 2019 and April 2021. Epidemiological and clinical characteristics regarding sex, age, family history of BC, pathologic tumor stage, tumor grade, and metastasis were included as baseline demographics for patients (unpublished records). Patients were diagnosed as at high risk for familial BC based on the criteria provided by the National Comprehensive Cancer Network guidelines. Controls, frequently matched to cases by average age, received routine mammography. The exclusion criteria for controls included abnormal mammography or prior personal history of any cancer. Family history of cancer was defined as self-reported BC in first-degree relatives. BC cases having other cancers or any negative histopathologic diagnosis besides BC were excluded (Figure 1).

Genotyping analysis

Genomic DNA was extracted from intravenous blood on ethylenediaminetetraacetic acid (EDTA) solution using the QIAamp DNA kit (Qiagen GmbH, Hilden, Germany). We performed TaqMan™ (Thermo Fischer Scientific, Waltham, MA) real-time polymerase chain reaction (PCR) analysis to genotype individuals for the selected SNPs of the rs2910164, rs11614913, and rs3746444 with the previously described oligonucleotides and probes [11] using a 7500 Fast-Dx Real-Time PCR System (Thermo Fischer Scientific, Waltham, MA). Negative controls and DNA samples were included in the assays. Some genotypes were validated by genotyping using the Genetic Analyzer 3500 (Thermo Fischer Scientific, Waltham, MA).

Statistical analysis

Hardy-Weinberg equilibrium (HWE) analysis was used to compare observed genotypes with expected genotypes in cases and controls for three loci, rs11614913 C/T, rs374644 A/G, and rs2910164 C/G, in terms of chi-squared (χ^2^) and P-values. Several inheritance models were analyzed, including codominant, dominant, recessive, overdominant, and additive, utilizing the SNPStats software (https://www.snpstats.net) (Institut Català d'Oncologia, Barcelona, Spain). Logistic regressions were calculated with odds ratios (ORs) and 95% confidence intervals (CIs) for genotypic distributions of cases and controls. The haplotyping and linkage disequilibrium (LD) between the examined miRNA markers are determined by the linkage disequilibrium (D) coefficient and the correlation coefficient between loci (r) pairs.

Protein-protein interaction (PPI) network

Using the miRNet database (https://www.mirnet.ca/), the potential protein-protein interaction (PPI) network was analyzed to predict functional interactions among miR-146a, miR-196a, miR-499, and other targeted genes.

## Results

Study population

**Figure 2:**
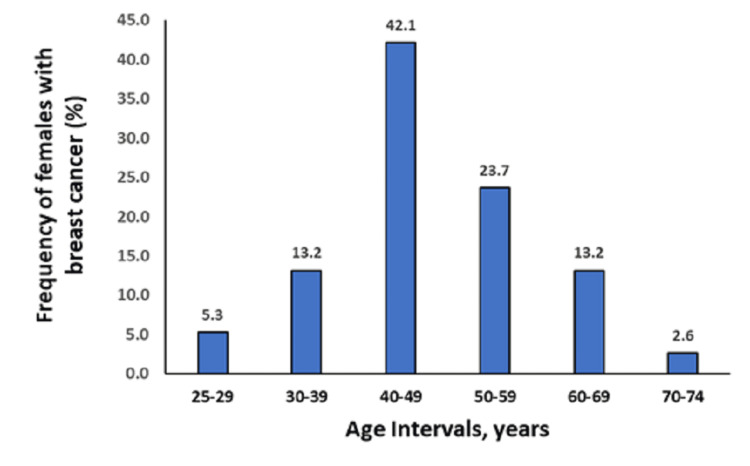
Age-specific breast cancer incidence in Saudi females

Seventy-six eligible female Saudi individuals with BC and 78 healthy females as a control group were enrolled. The mean age of patients was 47.5 ± 10.6 years, with no significant difference when compared with controls (t = 0.4; P = 0.7). The present study revealed the highest percentage of females with BC (42.1%) aged 40-49 (Figure 2). However, younger females with BC (aged ≤ 40) may have poorer prognoses than older females.

HWE of miRNA SNPs

Hardy-Weinberg equilibrium (HWE) was found at the analyzed SNPs in the miRNAs (P > 0.05). The genotypic distribution of the miR-146 rs2910164 C>G, miR-196a rs11614913, and miR-499 rs3746444 A>G were consistent with HWE in controls (χ^2^ = 2.80, P = 0.094; χ^2^ = 3.71, P = 0.0.06; and χ^2^ = 1.82, P = 0.178, respectively). In cases, the rs2910164 C>G and rs3746444 A>G deviated from HWE (P < 0.05), but the rs11614913 (χ^2^ = 2.56; P = 0.11) was consistent with HWE.

Allele frequencies

The miRNA regions rs2910164 C>G, rs11614913 C>T, and rs3746444 A>G SNPs were genotyped in 154 individuals: 76 females with BC and 78 controls using TaqMan™ real-time PCR assays. The allele frequencies showed that the ORs of the allelic variants were 1.37 (95% CI, 0.87-2.18; P = 0.177) for the rs2910164 C>G, 1.04 (95% CI, 1.61-1.76; P = 0.893) for rs11614913 C>T, and 0.87 (95% CI, 0.56-1.37; P = 0.557) for rs3746444 A>G SNPs (Table 1).

**Table 1:** Genotype and allele frequencies of selected SNPs in miRNA regions and their associations with breast cancer risk Bold numbers indicate statistically significant associations (P < 0.05) OR, odds ratio; CI, confidence interval; SNPs, single-nucleotide polymorphisms; miRNA, microRNA

Genetic model	Interactive genotype	Patients	Controls	Logistic regression	P-value
		n = 76	n = 78	OR (95% CI)	
miR-146a rs2910164 C>G					
Codominant	G/G	16 (21.1)	30 (38.5)	1	
	G/C	56 (73.7)	42 (53.9)	2.50 (1.2-5.17)	0.0135
	C/C	4 (5.3)	6 (7.7)	1.25 (0.31-5.09)	0.7553
Dominant	G/G	16 (21.1)	30 (38.5)	1	
	C/G-C/C	60 (79.0)	48 (61.5)	2.34 (1.11-4.8)	0.0197
Recessive	G/G-C/G	72 (94.7)	72 (92.3)	1	
	C/C	4 (5.3)	6 (7.7)	0.67 (0.18-2.46)	0.543
Overdominant	G/G-C/C	20 (26.3)	36 (46.1)	1	
	C/G	56 (73.7)	42 (53.9)	2.40 (1.22-4.73)	0.0113
Allele	G	88 (0.58)	102 (0.65)	1	
	C	64 (0.42)	54 (0.35)	1.37 (0.87-2.18)	0.177
miR-196a rs11614913 C>T					
Codominant	C/C	34 (44.7)	36 (46.1)	1	
	C/T	38 (50.0)	39 (50.0)	1.79 (0.37-8.74)	0.76
	T/T	4 (5.3)	3 (3.8)	1.05 (0.05-21.99)	
Dominant	C/C	34 (44.7)	36 (46.1)	1	
	C/T-T/T	42 (55.3)	42 (53.9)	1.67 (0.36-7.73)	0.51
Recessive	C/C-C/T	72 (94.7)	74 (96.2)	1	
	T/T	4 (5.3)	3 (3.8)	0.77 (0.04-14.38)	0.86
Overdominant	C/C-T/T	38 (50.0)	39 (50.0)	1	
	C/T	38 (50.0)	39 (50.0)	1.78 (0.39-8.17)	0.46
Allele	C	106 (0.70)	111 (0.71)	1	
	T	46 (0.30)	45 (0.29)	1.04 (0.61-1.76)	0.893
miR-499 rs3746444 A>G					
Codominant	A/A	28 (36.8)	24 (30.8)	1	
	A/G	28 (36.8)	33 (42.3)	6.15 (0.75-50.34)	0.19
	G/G	20 (26.3)	21 (26.9)	3.62 (0.40-33.12)	
Dominant	A/A	28 (36.8)	24 (30.8)	1	
	A/G-G/G	48 (63.2)	54 (69.2)	4.95 (0.71-34.27)	0.084
Recessive	A/A-A/G	56 (73.7)	57 (73.1)	1	
	G/G	20 (26.3)	21 (26.9)	1.31 (0.21-5.97)	0.89
Overdominant	A/A-G/G	48 (63.2)	45 (57.7)	1	
	A/G	28 (36.8)	33 (42.3)	2.99 (0.61-14.81)	0.17
Allele	A	84 (0.55)	81 (0.52)	1	
	G	68 (0.45)	75 (0.48)	0.87 (0.56-1.37)	0.557

Interactive genotypic distributions

As for genotypic distributions, significant associations were found for the rs2910164 C>G in most interactive models of inheritance: 2.50 (95% CI, 1.2-5.17; P = 0.0135) in the codominant model, 2.34 (95% CI, 1.11-4.8; P = 0.0197) in the dominant model, and 2.40 (95% CI, 1.22-4.73; P = 0.0113) in overdominant models (Table 1). The rs2910164 C/G heterozygous genotype showed overexpression in cases compared to controls (73.7% versus 53.9%; χ^2^ = 6.5; P = 0.011), but the homozygous rs2910164 G/G showed a significant protective effect (21.1% versus 38.5%; χ^2^ = 17.4; P = 0.019). The heterozygosity did not affect the risk to the BC in the rs11614913 (C>T) and rs3746444 (A>G). Thus, no association between the rs11614913 and rs3746444 in genotypes and BC risk was observed in the codominant, dominant, recessive, overdominant, or log-additive genetic models.

Haplotype association and linkage disequilibrium

Examining the miR-146a rs2910164 C/G, miR-196a2 rs11614913 C/T, and miR-499 rs3746444 A/G SNPs, we found that the C-T-G haplotype shows a statistically significant association with P = 0.0217 (Table 2). Besides, the correlation coefficients of linkage disequilibrium among the examined rs2910164, rs11614913, and rs3746444 SNPs were not in LD, and their associations were not statistically significant (P > 0.05).

**Table 2:** Haplotype frequency estimation of the examined miR-146a C>G, miR-196a A>G, and miR-499 A>G in female breast cancer (n = 154) Statistical analysis was based on logistic regression analysis. The bold number indicates a statistically significant P-value (P < 0.05)

Haplotype	miR-146a rs2910164 C>G	miR-196a rs11614913 C>T	miR-499 rs3746444 A>G	Total	Breast cancer, n = 76	Control, N = 78	Cumulative frequency
1	G	C	A	0.2263	0.2288	0.2269	0.2263
2	C	C	A	0.1672	0.1601	0.1705	0.3935
3	G	C	G	0.1657	0.1223	0.2355	0.5592
4	C	C	G	0.1439	0.1863	0.0787	0.7031
5	G	T	G	0.1297	0.1388	0.1065	0.8328
6	G	T	A	0.0877	0.0891	0.085	0.9205
7	C	T	A	0.0578	0.0747	0.0369	0.9783
8	C	T	G	0.0217	0	0.06	1

Protein-protein interaction network

**Figure 3:**
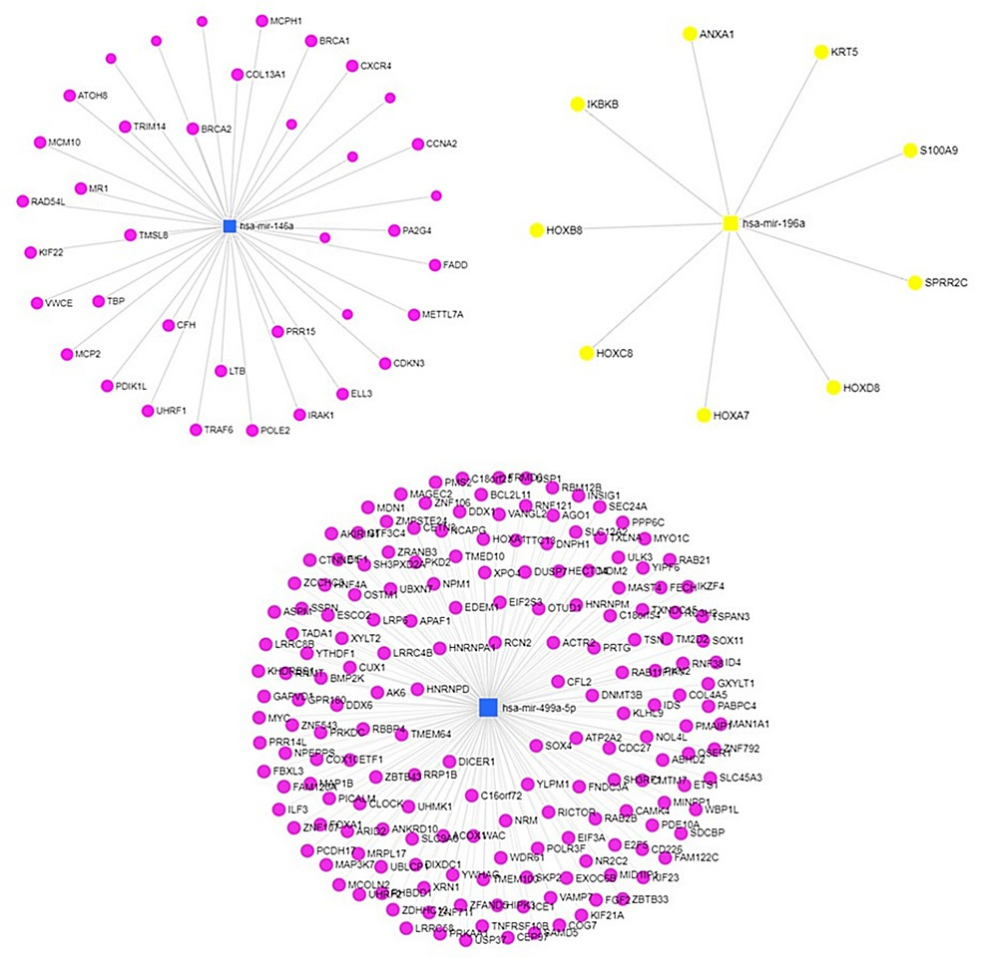
miRNA-target gene interaction network of differentially expressed miRNA constructed using the miRNet analysis (https://www.mirnet.ca/) miRNAs in squares, associations in edges, and target genes in nodes miRNAs: microRNAs

The results of miRNet analysis (https://www.mirnet.ca/) figured up strong associations of the examined miRNAs with related genes: 38 associations (edges) with miR-146a, 19 associations with miR-196a, and 181 associations with miR-499 present in the PPI network (Figure 3).

## Discussion

This hospital-based case-control study presents the first evidence that common miR-146a C>G (rs2910164), miR-196a C>T (rs11614913), and miR-499 A>G (rs3746444) may be used as candidate genetic markers for the risk of female BC in a western Saudi community. The heterozygosity of the rs2910164 C/G SNP shows a potential effect on the risk of BC, but the homozygous rs2910164 C/G reveals a protective effect on BC.

Several reports have shown no associations between BC risk and rs2910164 [16-22] and rs11614913 [18,19,23-25] in most diverse populations. On the contrary, the miR-164a rs2910164 was reported as susceptible to BC in 300 Pakistani females [26] (Tables 3, 4). The rs3746444 SNP has been reported to be associated with female BC in Chinese [16,27] and Iranians [28], except in South Americans [20] and Saudis ([29] and this study) (Table 5).

**Table 3:** Genotypes and allelic variants in miR-146a C>G in female breast cancer of different populations Bold numbers indicate a statistically significant P-value (P < 0.05)

Population	Cases (controls)	miR-146a C>G genotypes, cases (controls)			P-value	Variant allele, cases (controls)	P-value	Reference
		C/C	C/G	G/G		G		
Saudi Arabia	76 (78)	5.3 (7.7)	74 (54)	21 (39)	0.014	0.58 (065.)	0.18	This study
China	1,009 (1,093)	33 (33)	51 (50)	17 (17)	0.22	0.62 (0.71)	0.35	Hu et al., 2009 [16]
Italy	348 (155)	5 (4)	36 (38)	59 (58)	0.87	0.77 (0.77)	0.11	Pastrello et al., 2010 [17]
China	159 (298)	60 (112)	73 (154)	26 (32)	0.23	0.39 (0.37)	0.42	Deng et al., 2015 [18]
Indian	98 (99)	0 (0.0)	47 (39)	53 (60)	0.29	0.65 (0.72)	0.11	Bodal et al., 2017 [19]
South America	440 (1,048)	7.4 (9.0)	39 (38)	54 (54)	0.06	0.72 (0.73)	0.20	Morales et al., 2018 [20]
Southern Brazil	326 (411)	16 (21)	144 (148)	166 (242)	0.03	0.73 (0.77)	0.70	Brincas et al., 2020 [21]
Iranian	50 (50)	21 (21)	42 (47)	37 (32)	0.39	0.58 (0.55)	0.72	Siasi and Solimani, 2020 [22]
Pakistan	300 (300)	31 (14)	53 (42)	16 (44)	<0.0001	0.42 (0.64)	<0.0001	Ahmad et al., 2019 [26]

**Table 4:** Genotypes and allelic variants in miR-196 C>T in female breast cancer of different populations Bold numbers indicate statistically significant P-values (P < 0.05)

Population	Cases (controls)	miR-196a2 C>T genotypes, cases (controls)			P-value	Variant allele, cases (controls)	P-value	Reference
		C/C	C/T	T/T		T		
Saudi	76 (78)	44.7 (46)	50 (50)	5.3 (4)	0.76	0.30 (0.29)	0.893	This study
Indian	100 (100)	48 (64)	47 (35)	0.0 (0)	0.04	0.43 (0.59)	0.11	Bodal et al., 2017 [19]
Chinese	321 (290)	168 (185)	119 (88)	34 (17)	0.03	0.65 (0.52)	0.72	Qi et al., 2015 [23]
Irani	200 (200)	36 (26)	128 (160)	36 (14)	0.02	0.58 (0.55)	0.620	Nejati-Azar and Alivand, 2018 [24]
South American	441 (479)	181 (166)	209 (229)	36 (71)	0.002	0.28 (0.70)	0.583	Hoffman et al., 2009 [25]

**Table 5:** Genotypes and allelic variants in miR-499 A>G in female breast cancer of different populations Bold numbers indicate statistically significant P-values (P < 0.05)

Population	Number of cases (controls)	Genotypes of miR-499 A>G, cases (controls)			P-value	Variant allele, cases (controls)	P-value	Reference
		A/A	A/G	G/G		G		
Saudi Arabia	76 (78)	28 (24)	28 (33)	20 (21)	0.011	0.68 (0.75)	0.557	This study
Chinese	1,009 (1,093)	707 (816)	258 (248)	44 (29)	0.037	0.30 (0.28)	0.028	Hu et al., 2009 [16]
South American	440 (1048)	319 (772)	111 (254)	10 (22)	0.31	0.13 (0.30)	0.62	Morales et al., 2018 [20]
Chinese	321 (290)	152 (141)	117 (112)	52 (37)	0.34	0.66 (0.82)	0.35	Qi et al., 2015 [23]
Chinese	560 (583)	407 (463)	135 (109)	18 (11)	0.008	0.29 (0.11)	0.005	Dai et al., 2016 [27]
Iranian	43 (48)	39 (35)	39 (37)	11 (15)	0.029	0.39 (0.47)	0.047	Kabirizadeh et al., 2016 [28]
Saudi Arabia	100 (89)	30 (45)	62 (40)	8 (15)	0.001	0.78 (0.70)	0.18	Alshatwi et al., 2012 [29]

As for the genotypic distribution of the rs2910164 C>G, the significant association in our Saudi females with BC agrees with Southern Brazilian [21] and Pakistani [26] populations. Contrary to the lack of associations in Saudi females with BC, the miR-196a rs11614913 SNP showed susceptibility to BC in different populations, e.g., Indian [19], Chinese [23], Iranian [24], and South American [25]. In contrast, no associations were reported in diverse ethnics. Besides the present study, three reports have found associations between the Iranian [28], Chinese [16,27], and Saudi [29] populations. The stratification data of a meta-analysis showed a significant association between the has-miR-146a rs2910164 and cancer susceptibility in Asians but not in Caucasian populations [30]. Thus, it is probably believed that assessing the role of rs2910164 and rs11614913 SNPs in BC is essential for further large multicenter studies investigating different ethnic groups [18,25,31].

Additional observations suggested that rs2910164 and rs11614913 affected cancer risk in other organs, such as papillary thyroid carcinoma and hepatocellular carcinoma [31,32], and the rs11614913 SNP increased the risk for lung cancer [33]. Jazdzewski et al. [31] reported that the miR146a rs2910164 SNP affected the miR expression, contributing to the genetic predisposition to papillary thyroid carcinoma through tumorigenesis in somatic mutation. In non-small cell lung cancer survival, the rs11614913C allele was significantly associated with poor survival, indicating that it could have implications for cancer risk [23,25].

Still, findings have been inconsistent for different cancer types and participants from diverse populations regarding an association with specific cancers or malignancies. Ma et al. [34] reviewed the interaction of miRNAs with critical pathways such as tumor protein p53 (TP53), nuclear factor (NF)-kappa B, and beta-catenin pathways in colorectal cancer (CRC), a significant role in the regulation of epithelial-to-mesenchymal transition and the maintenance of cancer stem cell. Also, tumor suppressor miRNAs and oncomiRs supported their roles in CRC stem cells, epithelial-to-mesenchymal transition via different signalling pathways such as the Wnt/β-catenin activation, epidermal growth factor receptor (EGFR) pathway (transforming growth factor-beta {TGF-ß}), and the TP53 network [35]. Moreover, the overexpression of miR-146a has been linked to breast, pancreatic, and prostate cancers [18,26,30]. Furthermore, Peng et al. [36] found a strong association of the miR-196a2 with the lymph node metastasis of gastric cancer (P = 0.011) and a significant overexpression of miR-196a2 (CC) variant homozygote (P = 0.038).

Limitations of the study

Pinning down the miRNA SNPs for BC has been difficult because of the poor replication of studies. Firstly, several studies have been conducted in populations with admixed ethnicities, while we restricted our criteria to individuals from western regions of Saudi Arabia. Secondly, various studies included different miRNA SNPs inconsistent with HWE in controls or cases, which may have given rise to biased results of positive or negative associations. The present study focused on three polymorphic biomarkers associated with BC risk. Thus, we plan to conduct a panel of miRNAs to discover significant associations between BC and miRNAs in large sample sizes to strengthen our associations' validity.

## Conclusions

Emerging data highlight the essential role of miRNA biomarkers in BC risk among Saudi females. Despite lacking associations with the examined miRNAs, the heterozygous genotype rs2910164 C/G can identify at-risk females. More studies should be replicated using a panel of miRNA genes to discover significant associations with BC risk. BC prognostic biomarkers may be genetic polymorphisms in the miRNA flanking region. By combining polymorphisms with traditional epidemiological risk factors, it will be possible to identify individuals' risk factors within a few years. Biomarkers are promising for diagnosing, predicting, and monitoring treatment responses based on miRNAs. The overexpression of BC among young females aged 40-49 should be taken carefully while figuring out the global prognosis programs and treatment courses.
